# A Review on Locomotor Training after Spinal Cord Injury: Reorganization of Spinal Neuronal Circuits and Recovery of Motor Function

**DOI:** 10.1155/2016/1216258

**Published:** 2016-05-11

**Authors:** Andrew C. Smith, Maria Knikou

**Affiliations:** ^1^Interdepartmental Neuroscience Program, Northwestern University, Chicago, IL 60611, USA; ^2^The Graduate Center, City University of New York, New York, NY 10016, USA; ^3^Department of Physical Therapy, College of Staten Island, City University of New York, Staten Island, NY 10314, USA

## Abstract

Locomotor training is a classic rehabilitation approach utilized with the aim of improving sensorimotor function and walking ability in people with spinal cord injury (SCI). Recent studies have provided strong evidence that locomotor training of persons with clinically complete, motor complete, or motor incomplete SCI induces functional reorganization of spinal neuronal networks at multisegmental levels at rest and during assisted stepping. This neuronal reorganization coincides with improvements in motor function and decreased muscle cocontractions. In this review, we will discuss the manner in which spinal neuronal circuits are impaired and the evidence surrounding plasticity of neuronal activity after locomotor training in people with SCI. We conclude that we need to better understand the physiological changes underlying locomotor training, use physiological signals to probe recovery over the course of training, and utilize established and contemporary interventions simultaneously in larger scale research studies. Furthermore, the focus of our research questions needs to change from feasibility and efficacy to the following: what are the physiological mechanisms that make it work and for whom? The aforementioned will enable the scientific and clinical community to develop more effective rehabilitation protocols maximizing sensorimotor function recovery in people with SCI.

## 1. Introduction

There are more than 250,000 persons living with spinal cord injury (SCI) in the United States and several million worldwide. Injuries of the spinal cord occur mostly in young adults who then require life-long healthcare. The impaired function of spinal circuitry, the impaired processing of afferent input by the spinal circuits, and the decline in transmission of uninjured fibers are clear markers of the central nervous system's (CNS) pathophysiological state after SCI [1–5].

The understanding of spinal control of locomotion has improved significantly since the times of Thomas Graham Brown and Sir Charles Sherrington [6–8]. Complex models are currently developed to address the function of the spinal networks that give genesis to single limb and bilateral right-left neuronal interactions [9, 10], as well as their reorganization abilities following locomotor training in animal preparations [11]. Based on the observations on the spinal neural control of locomotion and recovery of locomotion in spinalized animals, body weight support (BWS) on a treadmill with as-needed manual assistance by therapists [12, 13] and BWS on a treadmill with robot-driven leg assistance [14] are utilized to improve locomotor ability of these patients. In this review, we will provide an in-depth discussion about the manner in which spinal neuronal circuits are impaired after SCI, how they reorganize after locomotor training, the possible neurophysiological mechanisms underlying such reorganization, and the functional consequences of locomotor-training-mediated neuronal plasticity.

## 2. Neuromodulation as a Window of CNS Function

Representative examples of neuronal activity modulation, recorded through surface electromyography (EMG) upon peripheral skin/nerve or transcortical stimulation, while walking in humans are found in three examples: the Hoffmann (H) reflex, motor evoked potentials (MEPs), and the polysynaptic flexor reflex. First, the H-reflex, which presents the spinal part of the stretch reflex bypassing the muscle spindle and the fusimotor activity that may influence the sensitivity of the Ia afferents, is a powerful tool to probe the efficacy of Ia afferents to monosynaptically depolarize alpha motoneurons, the excitability state of spinal interneuronal circuits/pathways, and spinal integration of sensory afferent feedback [15]. Second, MEPs, which are the result of spinal motoneuron activation following single-pulse transcranial magnetic stimulation (TMS), can be used to assess corticospinal tract excitability while walking in humans [16]. Third, stimulation of the skin at varying multiples of perceptual threshold of the foot or a pure sensory nerve (sural) can evoke short-latency, middle latency, or even long-latency responses in flexors and extensors that have specific regulatory effects on locomotion [17].

**Figure 1:**
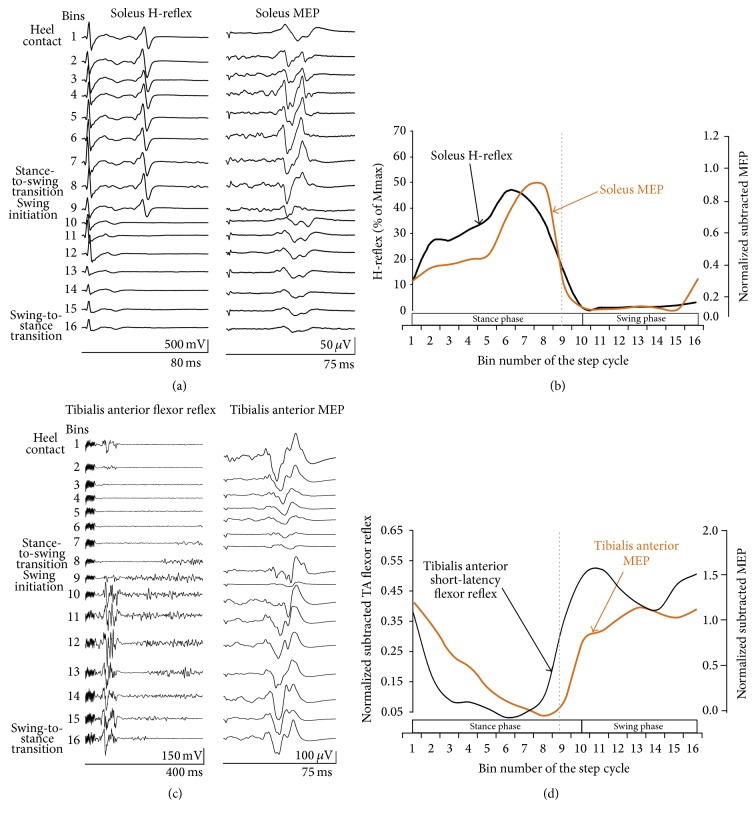
Modulation of neuronal activity while walking in uninjured humans. (a, b) Soleus H-reflexes and soleus motor evoked potentials (MEPs) amplitude at each bin of the step cycle while stepping on a motorized treadmill for single subjects (a) and for a group of healthy subjects (b). (c, d) Short-latency tibialis anterior (TA) flexor reflexes and TA MEPs amplitude at each bin of the step cycle while stepping on a motorized treadmill for single subjects (c) and for a group of healthy subjects (d). For the grouped data, for each bin of the step cycle, the soleus H-reflex was normalized to the maximal M-wave evoked 60–80 ms after the test H-reflex, and the short-latency TA flexor reflexes and soleus/TA MEPs were normalized to the maximum homonymous locomotor EMG having subtracted the control EMG (EMG without stimulation) at identical time windows and bins. Each step was divided into 16 equal bins based on the signal from the right foot switch. Bin 1 corresponds to heel contact. Bins 8, 9, and 16 correspond approximately to stance-to-swing transition, swing initiation, and swing-to-stance transition, respectively. Vertical dotted lines designate the stance-to-swing transition phase. Data adopted and modified from [2, 16, 21, 22].

In healthy humans, while stepping on a motorized treadmill, the soleus H-reflex amplitude increases progressively from midstance to late stance, decreases significantly at stance-to-swing transition, and remains depressed during the swing phase of gait (Figure 1(a)) regardless of the BWS level [18]. A gradually increasing H-reflex amplitude towards the end of the swing phase in healthy humans has also been reported (see [19, Figure 31]). A similar modulation pattern is also exhibited by the soleus MEP while walking. It increases progressively from early stance to midstance, reaching maximal amplitude at late stance, and is completely abolished during the swing phase with a gradually increasing MEP excitability at swing-to-stance transition (Figure 1(a)) regardless of the BWS level [16, 20]. Further, the short-latency tibialis anterior (TA) flexor reflex, evoked following innocuous stimulation of the skin over the medial arch of the foot, increases at heel contact, progressively decreases during the stance phase, and then increases throughout the swing phase in healthy humans while stepping on a motorized treadmill (Figures 1(c) and 1(d)), a pattern similar to that observed for the TA MEP (Figures 1(c) and 1(d)) [16, 21].

### 2.1. Spinal Reflexes and MEP Modulation

The amplitude modulation of soleus H-reflex, soleus MEP, and short-latency TA flexor reflex occurs largely in parallel with that of homonymous EMG activity [16, 21]. Because the soleus H-reflex remains depressed during the swing phase upon voluntary activation of the triceps surae, it is modulated in a similar manner to that observed while walking in absence of weight-bearing upon unilateral rhythmic leg movements, and the soleus MEP facilitation at swing-to-stance transition coincides with quiescent homonymous EMG [16, 22, 23], modulation of the soleus H-reflex and soleus MEP while walking cannot not be regarded simply as a sole reflection of background excitability changes of the motoneuron pool.

The soleus H-reflex amplitude modulation while walking can be partially ascribed to (1) presynaptic regulation of synaptic transmission from group Ia afferents to motoneurons and interneurons, (2) presynaptic regulation of GABAergic inhibition acting on dorsal root afferents, (3) phasic depolarization of group I afferents, and (4) tonic decrease in the excitability of the afferent fibers (animal data: [24–27]; human data: [28, 29]). Further, Ib facilitation [30, 31] and reciprocal Ia inhibition from flexor nerve afferents onto extensor motoneurons [32] also constitute spinal segmental mechanisms that contribute to the soleus H-reflex amplitude modulation at the stance and swing phases, respectively. Presynaptic inhibition of Ia afferent terminals and Renshaw cells acting on Ia inhibitory interneurons have also been documented [33]. The phase-dependent modulation of soleus and TA MEPs may be attributed to excitability changes of corticomotoneuronal cells [34], corticospinal volleys activating mutual reciprocal inhibitory interneurons [16, 35, 36], and cortically mediated ongoing changes in presynaptic inhibition of Ia afferents [37]. Excitatory and inhibitory interneurons in the motor cortex may contribute to MEP excitability changes while walking [38, 39]. The phase-dependent modulation pattern of the short-latency TA flexor reflexes can be partly attributed to amplitude modulation of presynaptic inhibition of cutaneous afferent volleys [25, 40].

For a phase-dependent modulation of soleus H-reflex, soleus/TA MEPs, and short-latency TA flexor reflexes to occur, locomotor neuronal networks need to be appropriately engaged at each phase of a step cycle, and thus these networks can depict both the physiological function and the underlying neuronal reorganization of spinal locomotor circuits in spinal-injured humans after repetitive step training.

## 3. Plasticity of Neuronal Activity after Locomotor Training

Following induction of SCI, animal studies have shown that locomotor training improves locomotor capacity beyond spontaneous recovery, full weight-bearing ability is prolonged, and improved locomotion persists up to 6 weeks after training stops [41, 42]. Similarly, in humans with SCI, locomotor training improves limb coordination, limb kinematics, step symmetry, walking speed, endurance, and balance [43–49], reduces systolic blood pressure and heart rate [50], improves respiratory function [51], and reduces inflammatory status [52]. Improvements in standing, walking, and respiratory capacity are likely due to plasticity of spinal interneuronal circuits. Below, we discuss evidence surrounding plasticity of neuronal activity after locomotor training in people with SCI.

### 3.1. Monosynaptic-Polysynaptic Motoneuron Responses While Walking

**Figure 2:**
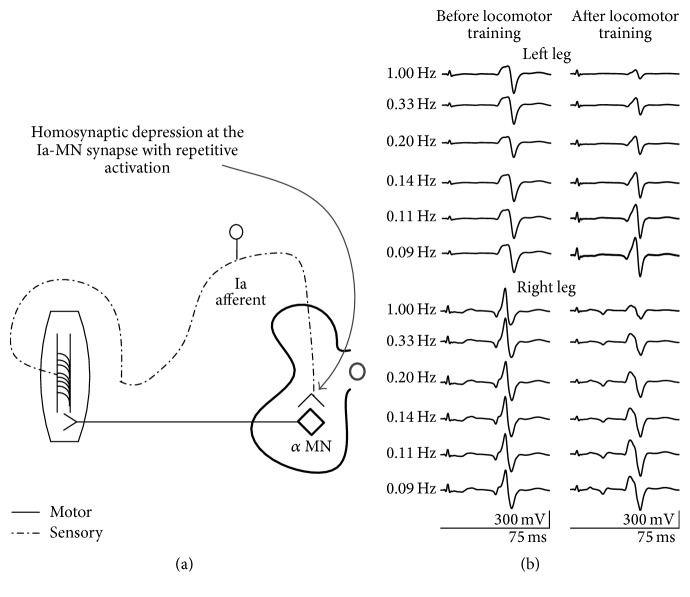
Functional reorganization of homosynaptic depression after locomotor training in SCI. (a) Schematic diagram of the soleus H-reflex homosynaptic depression exerted at Ia-motoneuron synapse with repetitive activation of Ia afferents. (b). Nonrectified waveform averages of soleus H-reflexes recorded at different stimulation frequencies from one AIS B patient before and after locomotor training for both legs. The soleus H-reflex amplitude exhibited a strong stimulation frequency-dependent depression after locomotor training. Data adopted and modified from [72].

In people with SCI, the soleus H-reflex modulation pattern while walking varies considerably between patients, from being relatively normal in some to being completely absent in others [2, 53, 54]. The most common abnormal patterns we have observed in people with SCI, regardless of the American Spinal Injury Association Impairment Scale (AIS), are lack of reflex depression during the swing phase and a disruption of sustained reflex excitability during the stance phase [54]. Similarly, the most common change we have observed after locomotor training regardless of the AIS is reestablishment of reflex depression during the swing phase that promotes reciprocal activation of ankle flexors and extensors [54]. Also importantly is our observation that reflex depression at mid-late swing was restored in two cases of motor complete SCI (AIS A-B) (see [54, Figure 2A]). We also observed that this neuronal reorganization was not distributed equally in the more impaired leg compared with the less impaired leg, as the soleus H-reflex during the stance phase was moderately decreased across all patients after locomotor training in the more impaired (right) leg compared to the less impaired (left) leg (compare Figures 2 and 3 in [54]).

**Figure 3:**
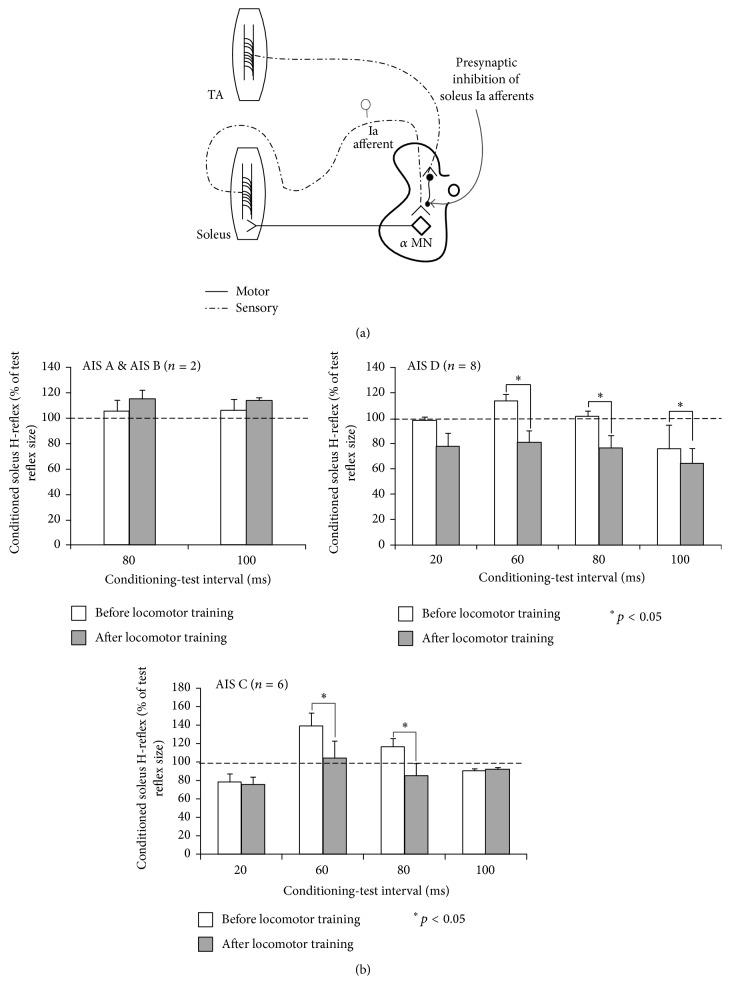
Functional reorganization of presynaptic inhibition of soleus Ia afferents after locomotor training in SCI. (a) Schematic diagram of the neuronal pathway of presynaptic inhibition of soleus Ia afferents. In this paradigm, presynaptic inhibition of soleus Ia afferents is induced by a conditioning afferent volley following common peroneal nerve stimulation at long conditioning-test (C-T) intervals. (b) Mean amplitude of the conditioned soleus H-reflex as a percentage of the unconditioned H-reflex recorded at each C-T interval tested before and after locomotor training from the right leg, grouped per AIS, in the seated position. ^*∗*^ *p* < 0.05 indicate statistically significant differences of the conditioned H-reflexes recorded before and after locomotor training. Data adopted and modified from [72].

An additional neuronal characteristic of SCI is the dominance of late long-lasting flexor reflexes over the short-latency flexor reflexes [1, 55–57]. While the late long-lasting flexor reflexes in human SCI have a similar interneuronal reorganization to that reported in acute spinal cats treated with L-DOPA (reviewed in [58]) and are due largely to the absent mutual inhibitory actions from early onto late flexor reflex interneuronal networks, their relative behavior signifies the altered interneuronal reorganization after injury. We recently reported that locomotor training in people with chronic SCI results in reappearance of short-latency TA flexor reflexes (see [28, Figure 3]), reduces the amplitude of long-latency TA flexor reflexes in the more impaired right leg (see [21, Figure 2]), increases the amplitude of long-latency TA flexor reflexes in the less impaired left leg (see [21, Figure 2]), and promotes a phase-dependent modulation of both short-latency and long-latency TA flexor reflexes during assisted stepping [21].

### 3.2. Spinal Inhibition

Impaired function of many different spinal inhibitory pathways has been implicated as one of the main causes of pathological movement and muscle tone after SCI, related to reduced GABAergic and glycinergic inhibitory neurotransmission/reception [59]. Physiological measures of neuronal activity, discussed below, strongly support that the main underlying neurophysiological mechanism of locomotor training is the return of the lost spinal inhibition in people with chronic SCI.

#### 3.2.1. Homosynaptic Depression

Homosynaptic (or low-frequency) depression is a form of presynaptic inhibition (Figure 2(a)) attributed mostly to a decrease in the amount of released neurotransmitters by the previously activated Ia afferents [60–62], depletion of releasable vesicles, failure of action potential conduction at axonal branches [63], decrease of presynaptic quantal size [64], and adaptation of exocytosis machinery [65]. Impaired function or completely absent homosynaptic depression in people with chronic SCI has been linked to stretch reflex hyperexcitability, clonus, and cocontractions due to altered or abnormal synaptic efficacy of afferent impulses [66–68].

**Figure 4:**
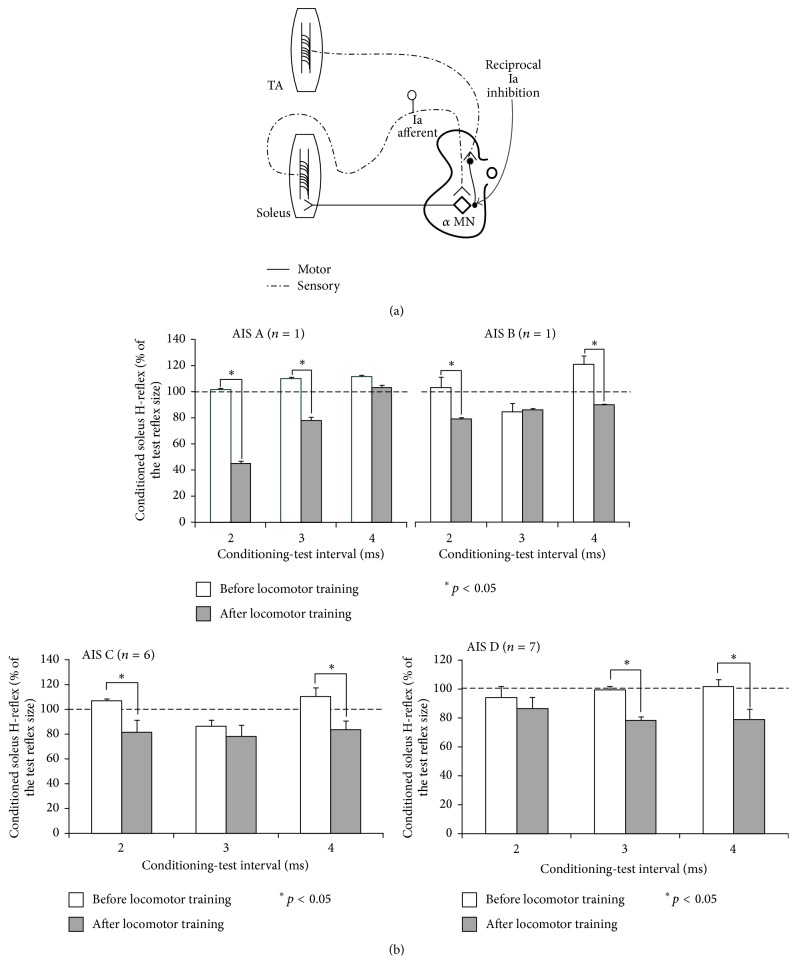
Functional reorganization of reciprocal Ia inhibition after locomotor training in SCI. (a) Schematic diagram of the neuronal pathway of reciprocal Ia inhibition mediated by a conditioning afferent volley induced by stimulation of the ipsilateral common peroneal nerve at short conditioning-test (C-T) intervals. (b) Mean amplitude of the conditioned soleus H-reflex as a percentage of the unconditioned H-reflex recorded at each C-T interval tested before and after locomotor training from the right leg, grouped per AIS, in the seated position. ^*∗*^ *p* < 0.05 indicate statistically significant differences of the conditioned H-reflexes recorded before and after locomotor training. Data adopted and modified from [90].

Limited evidence exists on the reorganization of homosynaptic depression in animals and humans. Homosynaptic depression was potentiated after passive exercise of complete spinal transected rats [69], after 10 locomotor training sessions in one SCI person capable of ambulation [70] and after cycling in one person with spastic tetraplegia [71]. We recently reported that repetitive locomotor training restores soleus H-reflex homosynaptic depression, but we found significant differences among patient groups [72]. In summary, we found that soleus H-reflex homosynaptic depression was restored in two people with motor complete SCI in both right and left legs, and it became stronger after training in the more impaired right leg compared to the less impaired left leg regardless of the AIS (see [72, Figure 4]). Last, we found that, in cases where some homosynaptic depression was present before training, locomotor training further potentiated the soleus H-reflex homosynaptic depression (see [72, Figure 4]). In Figure 2(b), representative examples of this neuronal organization are indicated from one person with AIS B who received 53 locomotor training sessions. These recordings clearly indicate that the soleus H-reflex amplitude exhibited a strong stimulation frequency-dependent depression after locomotor training even in cases when descending control is greatly impaired or absent [72].

#### 3.2.2. Presynaptic Inhibition

The synaptic efficacy of afferent volleys before they reach their target neurons can be adjusted by presynaptic inhibition (Figure 3(a)). Methods have been developed to probe presynaptic inhibition exerted only at Ia afferent terminals [71]. This is because only Ia afferents have monosynaptic projections to motoneurons and separation from motor fibers based on stimulation intensities and respective thresholds is possible. Presynaptic inhibition was originally described in the cat by Frank and Fuortes [73], is associated with primary afferent depolarization (PAD), is mediated by axoaxonic synapses [74], and involves modulation of transmitter release at the Ia-motoneuron synapse by means of GABA_A_ receptors, which consequently increase the efflux of Cl^−^ ions and produce depolarization of the afferent terminals [75].

Presynaptic inhibition is modulated in a phase-dependent manner during fictive and real locomotion in animals, including humans [25, 28, 76, 77], and accounts to a great extent for the differential soleus and quadriceps H-reflex amplitude modulation while walking in uninjured humans [29, 78]. Functionally, increased presynaptic inhibition of the soleus Ia excitatory feedback may be needed to prevent excessive activation of ankle extensor motoneurons at mid-to-late stance phases (causing a stiff gait), while decreased presynaptic inhibition of the quadriceps Ia excitatory feedback at early stance prepares the knee joint to accept loading. The soleus H-reflex facilitation following femoral nerve stimulation at group I threshold is exerted from quadriceps afferents onto soleus motoneurons via monosynaptic connections, and increases or decreases of this facilitation have been ascribed to changes in the ongoing presynaptic inhibition [79]. The excitatory influence of Ia afferents onto synergistic muscles, as is the case with quadriceps afferents acting onto soleus motoneurons, is also known as heteronymous Ia facilitation. In people with traumatic SCI, the increased heteronymous Ia facilitation supports decreased presynaptic inhibition [80]. The complete disappearance of presynaptic inhibition of Ia afferent terminals of the flexor carpi radialis H-reflex, elicited by electrical stimuli applied to the nerve supplying antagonistic muscles at long conditioning-test intervals, in two patients with tetraplegia due to a spinal cord lesion at C5-C6 [81], supports further abnormal premotoneuronal control after SCI. It has also been shown that the level of presynaptic inhibition declines over time after SCI [66]. The decrease of presynaptic inhibition after SCI is likely related to impaired function of the descending pathways that ensure suppression of inhibitory interneurons transmitting cutaneous inhibition of first-order PAD interneurons [82].

We recently reported that presynaptic inhibition of soleus Ia afferents, assessed as the amplitude of the conditioned soleus H-reflex by excitation of antagonistic group I afferents at long conditioning-test intervals in the seated position [15], was reorganized in motor incomplete SCI (AIS C-D) but not in motor complete SCI (AIS A-B) after locomotor training (Figure 3(b)) [72]. We also found that, during assisted stepping, the modulation of presynaptic inhibition occurred at different phases of the step cycle before training when compared to that observed after training [72], and this change was comparable to the modulation pattern we have reported for uninjured human subjects during assisted stepping [77]. Reorganization of presynaptic inhibition can partly account for the return of the physiological soleus H-reflex amplitude modulation while walking after locomotor training found for the same patients [54].

#### 3.2.3. Reciprocal Ia Inhibition

The neuronal pathway from the large muscle spindle (Ia) afferents to antagonistic alpha motoneurons is the most known and well-studied spinal inhibitory pathway in the mammalian CNS (Figure 4(a)), described originally by Lloyd [83–85], with vestibulo-, cortico-, and rubropropriospinal tracts and cutaneous and flexor reflex afferents to affect transmission in the Ia interneurons and their subsequent synaptic inputs onto motoneurons [86]. Ia afferent-mediated reciprocal inhibition is effective in blocking antagonist motoneuron activation at birth in hemisected spinal cord preparations and in humans when rhythmic motor programs have not been developed, used, or stored [87, 88]. A high specificity of neuronal connections from quadriceps Ia afferents to posterior biceps-semitendinosus motor neurons is reported at birth in mice [89].

The functional significance of reciprocal Ia inhibition is apparent when one considers that this neuronal pathway operates only between flexor and extensors and not between abductors and adductors [91]. Thus, the role of reciprocal Ia inhibition in the alternating activation of flexors and extensors during locomotion might be to eliminate excitatory effects during the passive (swing) phase of the step cycle and remove the enduring Ia excitation during the shifts between flexion and extension phases [92]. Recordings from Ia inhibitory interneurons during fictive locomotion in complete spinally transected cats showed that hyperpolarization of extensor alpha motoneurons during the swing phase is directly related to their activity [93–95], largely determined by intraspinal rhythmic processes [96].

SCI in humans is associated with pathologic changes of reciprocal Ia inhibition, with alterations reported in strength, timing, and modulation at rest, during contraction, and while walking [97–101]. Reciprocal facilitation is related to poor motor recovery of legs, while stronger reciprocal inhibition is linked to less spasticity (1 and 2 on the Ashworth score) [102].

In a group of people with SCI, we studied to what extent reciprocal Ia inhibition of soleus motoneurons, assessed as the soleus H-reflex amplitude conditioned by excitation of TA group I afferents at short conditioning-test intervals while seated [15], is restored after locomotor training. We found that reciprocal facilitation was replaced by reciprocal inhibition regardless of the AIS level in the seated position (Figure 4(b)) [90]. However, during assisted stepping the changes were not uniform across AIS patients, because we found that reciprocal Ia inhibition recovered at a greater level in AIS C than in AIS D after locomotor training (see [90, Figure 2]). Reciprocal inhibition was profoundly decreased during the stance phase and increased during the swing phase in AIS C after locomotor training, while, in AIS D, reciprocal inhibition was mostly decreased (see [90, Figure 2]). The reduced amounts of reciprocal inhibition in AIS D can explain the lack of full soleus H-reflex depression during the swing phase we observed in these patients. It is possible that more training sessions or more intense training (i.e., more steps/session) [103] may be required to increase the amount of reciprocal inhibition in some patients with SCI.

#### 3.2.4. Nonreciprocal Ib Inhibition

**Figure 5:**
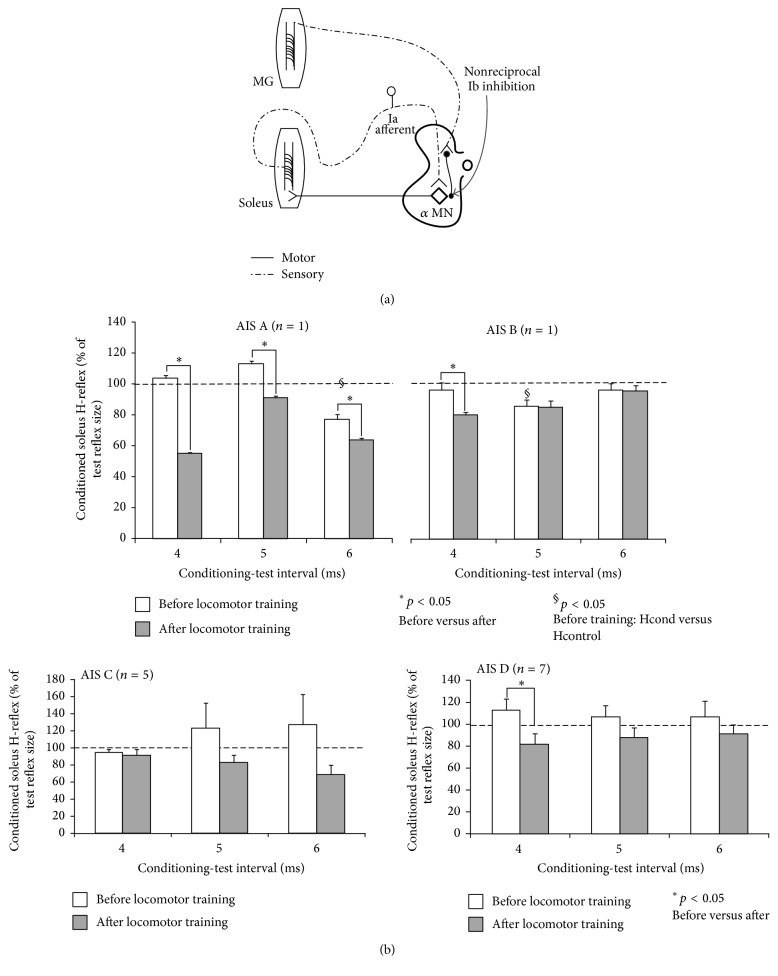
Functional reorganization of nonreciprocal Ib inhibition after locomotor training in SCI. (a) Schematic diagram of the neuronal pathway of nonreciprocal Ib inhibition mediated by a conditioning afferent volley induced by stimulation of the ipsilateral medialis gastrocnemius nerve at short conditioning-test (C-T) intervals. The facilitatory locomotor Ib pathway is not indicated. (b) Mean amplitude of the conditioned soleus H-reflex as a percentage of the unconditioned H-reflex recorded at each C-T interval tested before and after locomotor training from the right leg, grouped per AIS, in the seated position. ^*∗*^ *p* < 0.05 indicate statistically significant differences of the conditioned H-reflexes recorded before and after locomotor training. Data adopted and modified from [90].

The views pertaining to the functional role of Ib afferents (Figure 5(a)) have changed substantially from a simple autogenic protective reflex response to the more complicated view that these afferents continuously provide information about the amplitude of muscle contraction. Ib interneurons that mediate such information are widely distributed, reaching almost all motoneuron pools of the ipsilateral limb [104]. Ib interneurons can participate in alternative pathways allowing for excitation or inhibition depending on the selected subpopulation of interneurons [105] and receive extensive convergence from other afferents and descending tracts [106]. It is well established that Ib afferents participate in a reflex reversal during fictive locomotion in decerebrate cats, known as locomotor-related group I excitation, which utilizes a different circuit organization compared to that observed at rest and is transmitted through the extensor half centre [107, 108]. These Ib locomotor excitatory interneurons are located in the intermediate zone in mid to caudal parts of the lower lumbar spinal cord [30]. In summary, group I (mainly Ib) afferents of ankle extensors shape the amplitude, duration, and timing of ipsilateral extensor activity and depending on the timing that excitation occurs they can increase the activity of extensor motoneurons at the stance phase, initiate extension, and terminate or delay flexor bursts in the ipsilateral hind limb [107, 109–112]. A similar facilitatory locomotor group I pathway also exists in humans [113], with Ib inhibition decreasing while loading and reversing to excitation while walking [31].

In people with chronic spinal cord lesions, conflicting evidence exists on this pathway, as nonreciprocal Ib inhibition is reported to be either physiologic or pathologic at rest and during assisted stepping [114–116]. Indeed, we recently reported the presence of short-latency soleus H-reflex depression following medialis gastrocnemius (MG) nerve stimulation at short conditioning-test intervals (attributed mostly to Ib inhibition) in two persons with AIS A and AIS B while seated (Figure 5(b)) [90]. In addition, locomotor training potentiated the preexisting Ib inhibition at rest in AIS A, AIS B, and AIS C (Figure 5(b)), but during assisted stepping we found that the reorganization was different for AIS C and AIS D [90]. In general, changes in Ib inhibition were noted mostly during the swing phase in AIS C patients, while in AIS D patients Ib inhibition was increased at midstance [90]. While these findings are consistent with the reduced short-latency group I inhibition of synergists at the stance phase of walking in healthy humans and during fictive locomotion in spinal animals [113, 117], locomotor training did not induce, as expected, an extra facilitation of soleus motoneuron responses by group Ib afferents during the stance phase. Strengthening Ib polysynaptic excitation with locomotor training during the stance phase of walking may require more training sessions, more steps per session, more body loading, greater allowance for manageable errors, and/or training at different levels of environmental constraints [103, 118].

### 3.3. Alpha Motoneurons

Altered excitability of spinal neurons is considered a key pathophysiological event after an injury to the spinal cord. The monoamines serotonin and norepinephrine, which are released from pathways originating in the brainstem, substantially modulate spinal motoneuron excitability. Activation of monoamine receptors enhances intrinsic low-voltage-activated persistent inward currents (PICs) that produce plateau potentials and self-sustained firing in both the somata and dendrites, also regulating the gain of the motoneuron pool [119–123]. PICs amplify both synaptic excitation and inhibition, are critical for the dynamic transformation of synaptic inputs, and provide a sustained excitatory drive that allows motoneurons to fire repetitively following a brief synaptic excitation [124–126]. Inhibitory synaptic inputs can exert considerable control over alpha motoneuron discharge by regulating intrinsic PICs activation/deactivation [127].

Despite the lost or reduced brainstem-derived serotonin with chronic SCI, PICs are enhanced due to compensatory upregulation of constitutively active 5-HT_2_ receptors [128]. PICs that drive self-sustained firing in motoneurons have been related to the development of muscle spasms and hyperreflexia to nonnoxious stimuli and clonus [129]. Additionally, the voltage threshold of slow motoneurons changes, axonal conduction velocity, and rheobase current increases, afterhyperpolarization duration decreases [130, 131], short pulse current threshold increases [131], and resting threshold and resting membrane potential decrease [131, 132]. Further, spinal cord transection leads to changes in the rhythmic firing patterns of motoneurons in response to injected currents [132]. In people with SCI, alpha motoneuron PICs and associated self-sustained firing facilitated the firing of motor units during the prolonged muscle spasms that can continue for many seconds, even minutes, at very low discharge rates [133].

Evidence from animal studies suggests that intrinsic properties of motoneurons recover after locomotor training. Motoneurons of trained rats have lower hyperpolarized resting membrane potentials, decreased spike trigger threshold levels (membrane potential at which an action potential is triggered), increased amplitudes of after hyperpolarization (reflecting a decrease in membrane excitability) [134–136], stabilized dendritic tree structure of motoneurons [137], altered synaptic inputs from the spinal white matter [138], and a soma size and Na^+^, K^+^, and ATPase activity similar to uninjured animals [139]. The duration of training is critical in changing the intrinsic properties of motor neurons, as 3 weeks of training does not restore their electrical properties [140].

Direct changes in the electrical and biophysical properties of motor neurons in SCI patients following locomotor training are difficult to document given the methodological limitations in human studies. However, the amplitude of monosynaptic motoneuron responses at different stimulation intensities along with excitation thresholds can help us draw conclusions on these characteristics. The amplitude and stimulation threshold intensities of the soleus monosynaptic motoneuron responses are not adjusted in untrained SCI patients in the supine and standing positions compared to those observed in uninjured subjects [18]. We found that these parameters were remarkably modified in a body position-dependent manner that depended largely on the leg motor impairment after locomotor training [141]. The maximal H-reflex (Hmax) size was decreased after training while seated and while standing in AIS A and AIS B subjects [141]. The soleus H-reflex size, from the onset of the recruitment curve until its maximum amplitude, was decreased in the right leg in AIS D and in the left leg in AIS C while seated and was increased while standing in both legs in AIS C but not in AIS A, AIS B, and AIS D [141]. Further, the stimulus corresponding to H-threshold, 50% Hmax, and Hmax was increased in AIS D, in whom the reflex excitability was decreased in the right leg while seated after training [141, Table 1]. This means that, after locomotor training, more stimulation intensity is required to activate the most excitable Ia afferent fibers that subsequently depolarize the lower threshold (most excitable) soleus motoneurons. The stimulus corresponding to H-reflex threshold expresses the number of active motoneurons or the spinal excitability level, which reflects the balance of excitatory and inhibitory inputs acting on the motoneuron pool [142]. The decreased spinal reflex excitability with the concomitant increased soleus H-reflex threshold indicates that motoneuron excitability was altered along with the excitability level of Ia afferents.

The increased soleus H-reflex excitability we observed in AIS C subjects while standing after training, compared to that observed before training [141], may enhance ankle stability and thus contribute to an improved leg function while standing. It is known that in uninjured humans, while standing, Ib inhibition exerted from MG to soleus motoneurons is decreased, presynaptic inhibition of soleus Ia afferents is increased, and reciprocal inhibition is decreased when compared to that observed while seated [31, 143, 144]. Thus, both presynaptic inhibition and Ib facilitation after locomotor training may reinforce H-reflex excitability while standing, promoting weight-bearing in people with motor incomplete SCI. The neuronal activity changes we have recently reported after repetitive locomotor training in people with chronic motor complete and incomplete SCI [21, 54, 72, 90, 141] are summarized in Table 1 based on body position, motor task, and AIS. These changes can be summarized as follows: (1) monosynaptic motoneuron responses are adjusted in a body position manner, (2) soleus H-reflex phase-dependent modulation is restored, (3) soleus H-reflex homosynaptic depression is restored regardless of AIS, (4) presynaptic inhibition of the soleus Ia afferents evoked by a conditioning stimulus recovers only in AIS C and AIS D, (5) reciprocal Ia inhibition from flexor group I afferents on soleus motoneurons is absent before training and returns regardless of AIS after training, (6) Ib inhibition from MG group I afferents on soleus motoneurons is present before training and is increased after training in AIS A, AIS B, and AIS D, and (7) short-latency flexor reflexes reappear and both short- and long-latency flexor reflexes are modulated in a phase-dependent manner [21, 54, 72, 90, 141].

## 4. Recovery of Motor Activity after Locomotor Training in SCI

**Figure 6:**
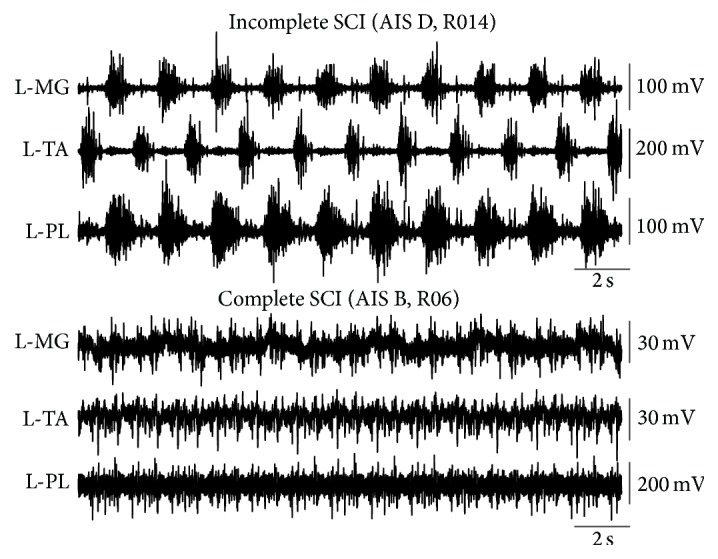
Motor activity after locomotor training in incomplete and complete SCI. Nonrectified electromyographic (EMG) activity from 10 consecutive steps of medialis gastrocnemius (MG), tibialis anterior (TA), and peroneus longus (PL) muscles from the left legs in one motor incomplete SCI subject (AIS D) and in one motor complete SCI subject (AIS B) during assisted stepping after locomotor training. Note in subject R014 that MG and PL occur in a reciprocal pattern with the TA, but distinctive EMG bursts are absent in subject R06.

Motor output can be viewed, without excluding the descending pathways, as the net result of function of motor neurons and interneurons at multisegmental spinal levels. Based on our latest completed locomotor trial in people with SCI and other studies, the changes in motor function can be summarized as (1) increase in peak EMG amplitudes of ankle muscles and decrease in peak EMG amplitudes of medial hamstrings and hip adductor gracilis muscles in the more impaired right leg (medial hamstrings and hip adductor gracilis muscles are known to contribute primarily to the spastic gait pattern and to the pathological leg spasticity pattern at rest) [72, Figure 5B], (2) restoration of biphasic EMG activity (when a muscle contracts in more than one phase within a single step cycle) (see [72, Figure 5A]), (3) onset changes of EMG activity while stepping, (4) reduced cocontraction levels between ankle and knee antagonistic muscles (see [72, Figure 5C]), (5) improvements in the alternating activity of the same muscle between the left and right legs, and (6) reduced EMG clonic activity of ankle extensors at rest and on the treadmill [54, 72, 145, 146]. However, locomotor training does not restore motor activity similarly in complete and incomplete SCIs. An example of an episode of muscle activity during assisted stepping after locomotor training is shown in Figure 6. The EMG bursts clearly indicate that the ankle antagonistic muscles were activated in a reciprocal pattern after locomotor training in the incomplete SCI subject (AIS D, R014), while a complete absent phase-dependent activity is evident in the complete SCI subject (AIS B, R06) after 53 locomotor training sessions (Figure 6). The lack of distinguished antagonistic EMG bursts with clear onset and offsets in the person with motor complete SCI after locomotor training clearly supports pronounced differences between recovery in animals and humans [147], and thus we need to be cautious when animal data are translated to humans.

The profound changes in motor activity after locomotor training in motor incomplete SCI coincided with changes in gait parameters. The BWS required while stepping was decreased by an average of 55%, the gait speed was increased by 58%, and the leg guidance force by the robotic exoskeleton was decreased by 43% [72]. Furthermore, in the motor incomplete subjects, locomotor training improved their lower extremity motor scores, assessed manually by a physical therapist, with the more impaired right leg improving by 10% and the left leg improving by 6.4% [21], a motor improvement also reported for cervical and thoracic AIS D patients [148].

## 5. Pathways and Circuits Underlying Neuronal and Motor Plasticity after Locomotor Training

The CNS adapts and reorganizes continuously based on motor experience and use. This* natural* reorganization is the result of physiological, anatomical, and functional neuronal changes along the neuroaxis (cortex, cerebellum, spinal cord, and nerve axons) [149]. After an injury to the spinal cord, neuronal reorganization occurs that eventually results in neural circuits/pathways with altered properties and functions. The findings on the neuronal activity changes in animals and in humans detailed in the above paragraphs support that locomotor trainingcan promote* functional* neuronalreorganization [150]. A major drive to the neuronal reorganization after locomotor training is reinforcement of activity-dependent sensory feedback from receptors (including but not limited to plantar mechanoreceptors and hip proprioceptors) that can adjust the operation of the CPG [11, 151, 152].

Sources for neuronal activity changes in people with SCI after locomotor training could include modifications in the intrinsic properties and function of the somata and dendrites of neurons, excitability profile of motoneuron pools, excitability thresholds of muscle afferents, modulation of EPSPs from afferents, and modifications on the descending control of spinal reflex networks involving synaptic and nonsynaptic mechanisms. These changes most likely occur simultaneously at differing strengths during the course of locomotor training, while adjustments made to the BWS, treadmill speed, and leg guidance force during the course of training [54] affected the reorganization of spinal neuronal pathways integrating information about body loading and muscle stretch.

The restored soleus H-reflex depression during the swing phase in motor complete SCI we observed after locomotor training points towards three directions: (1) the soleus H-reflex depression during the swing phase cannot be attributed solely to reciprocal Ia inhibition between ankle antagonistic muscles because the physiological supraspinal control of Ia inhibitory interneurons (animal: [98, 153, 154]; human: [98, 155]) is greatly impaired in AIS A and AIS B, (2) reciprocal Ia inhibition can become functional after locomotor training even when descending control is impaired, and (3) functional behavior of reciprocal Ia inhibition is not depicted well in the EMG bursts during assisted stepping (see EMG bursts of AIS B subject in Figure 6). While all of these directions entail limitations with respect to the relevant contribution of reciprocal inhibition to the reflex depression during the swing phase of gait, it is possible that this change represents the capacity of intrinsic properties of the spinal cord to alter rhythmic motor activity after training [156].

Differences between right-left leg soleus H-reflex modulation changes suggest that the connections made by commissural spinal cord interneurons to motoneurons [156, 157] might have been affected differently by locomotor training in some patients compared to others. Commissural interneurons interact with 5-HT and GABA systems, form excitatory and inhibitory connections onto contralateral motoneurons at latencies consistent with monosynaptic and polysynaptic pathways, are under descending influence, and support locomotor rhythm generation in response to brainstem stimulation [157–163]. Further, midline lesions and photoablation affecting the axons of these neurons eliminate rhythmic ventral root bursting, alter the symmetry of ventral root bursts, and can eliminate rhythmic bursting [163], supporting the contribution of commissural interneurons to rhythmogenesis.

In healthy humans, ipsilateral posterior tibial nerve stimulation or knee extension joint rotation produces inhibition in both the contralateral soleus motoneurons and the reflex responses in the contralateral biceps femoris muscle, both being modulated according to the phase of walking [164–167]. Crossed postsynaptic inhibition in contralateral soleus motoneurons from ipsilateral groups I and II afferents at short latencies (3–7 ms), similar to those reported for the feline spinal cord, has recently been described for humans [168]. Further, activation of contralateral hip proprioceptors results in ipsilateral soleus H-reflex depression [169]. Taken altogether, differences between right-left leg H-reflex changes during the stance phase may thus represent plastic changes of commissural interneurons, but it is evident that there is a need for in-depth exploration of the physiological changes of commissural interneurons in people with SCI after locomotor training.

Changes in presynaptic and postsynaptic inhibition after locomotor training may be related to changes in the strength of the depolarization of muscle afferents [24, 170, 171] or may be the result of transformations in the intrinsic properties of spinal neurons and afferents after locomotor training. For example, in anesthetized chronic spinal cats there is an overall increase in Ia excitatory postsynaptic potentials (EPSPs) in ankle extensor motoneurons [172]. Plantar mechanoreceptors interact with presynaptic inhibitory interneurons, in humans at rest and in spinalized cats during fictive locomotion [54, 173]. Additionally, plantar mechanoreceptors evoke a phase-dependent modulation of primary afferent depolarization [24, 25], alter their effects on spinal motoneurons in spinalized cats after step training [173], and normalize the function of monosynaptic spinal reflexes while stepping in untrained spinal cord-injured patients [174]. Changes in the intrinsic properties of spinal neurons after locomotor training are supported by the increased density of the glycinergic axonal terminals and decreased size of both glycinergic and GABAergic axon terminals in complete spinal trained transected rats compared to nontrained transected rats [175]. Because plasticity of the glycinergic system, which mediates inhibitory neurotransmission, occurs independently of supraspinal influence [176] and reciprocal Ia inhibition was potentiated after training in complete SCI at rest but recovery while stepping varied between patients, direct descending inputs on Ia inhibitory interneurons may not be a key source for neuroplasticity. However, this may be required for long-term support of inhibitory synaptic transmission and regulation of the depth of reciprocal Ia inhibition during locomotion.

The aforementioned are possible physiological changes in complete SCI, but the neuronal reorganization is more complex in incomplete SCI because neuronal structures above the lesion site might adapt the function and behavior of spinal neuronal circuitries known to control locomotor activity through remnant descending pathways. In incomplete SCIs, the plasticity of uninjured fibers plays an important role in functional recovery. In cats and monkeys, as little as 25% of remaining white matter tracts can allow for recovery of voluntary locomotor ability [177, 178], and a similar observation was found in humans following partial spinal cord transection to provide cancer pain relief [179]. Animal studies provide substantial direct evidence that, following a hemisection injury to the corticospinal tract, transected fibers sprout into cervical gray matter to communicate with propriospinal interneurons, whose propriospinal neurons then relay the motor command to distal lumbosacral motoneurons [180–185]. In rats, this corticopropriospinal connection can be enhanced pharmacologically [186] and with locomotor treadmill training [187]. Considerable indirect evidence suggests that this pathway is preserved in humans [188–192] and may be probed utilizing TMS and peripheral nerve stimulation [192, 193]. Future research on this pathway in humans with SCI and the effects of locomotor training is warranted.

## 6. Functional Consequences of Neuronal and Motor Plasticity

A question that arises is as follows: to what extent is plasticity of neuronal activity related to improvement of motor function in SCI patients? Although improvements in gait parameters were noted over the course of training, overground walking ability assessed by the 6-min walk test, and the number of sit-to-stand repetitions completed within 30 s, and the time needed to rise from a chair, walk for 3 m, and return to the chair were not improved after locomotor training in AIS C and AIS D [54]. Lack of changes in these walking ability variables could be related to (1) number of training sessions per participant, (2) small number of participants, (3) existence of nonresponders within the group, and (4) the fact that the 6-min walk test may not be sensitive enough to detect improvements in quality of walking of patients with SCI [194]. It may be also the case that the benefits seen with robotic-assisted treadmill training did not carry over into the task-specific overground testing of the 6 min walk test. However, previous literature involving locomotor training with as-needed manual assistance or with robotic-driven leg assistance in motor incomplete SCI demonstrated improvements in the walking index for SCI version II (WISCI-II) scale, overground walking speed, Berg balance scores, and 6 min walk test [146, 195–198]. Further, locomotor training improves lower extremity motor scores in both motor complete and motor incomplete SCIs [199, 200]. Taken altogether and including gaps in the literature, it is apparent that the time course of neuronal plasticity with corresponding motor recovery needs to be established.

## 7. Concluding Remarks

Locomotor training of persons with clinically complete, motor complete, or motor incomplete SCI induces reorganization of spinal neuronal networks that coincides with improvements in motor activity and decreased pathophysiological phenomena of the spasticity syndrome. However, to maximize recovery of motor function in patients with SCI, we need to utilize both established (i.e., locomotor training, spinal cord stimulation) and contemporary (i.e., brain controlled intraspinal microstimulation) technologies/interventions simultaneously and change the focus of our research questions from “feasibility” and “efficacy” to “what are the physiological mechanisms that make it work?” and “for which category of patients?” Additionally, while we need to better understand the physiological changes underlying locomotor training, especially of the uninjured fibers, research efforts should concentrate on providing strong scientific evidence when more than one intervention is utilized concomitantly. Over the course of treatment, physiological signals can be used to (1) probe recovery, (2) develop algorithms that may be used to define the approach of locomotor training for each patient, and (3) predict functional recovery. These approaches will enable the scientific and clinical community to develop more effective rehabilitation protocols.

## Figures and Tables

**(a):** 

Neuronal activity	AIS A/B		AIS C		AIS D	
	Seated	Stepping	Seated	Stepping	Seated	Stepping
Soleus H-reflex phase-dependent modulation	NA	↓ stance phase^*∗*^ ↓ swing phase^*∗*^	NA	↓ swing phase^*∗*^	NA	↓ swing phase^*∗*^
Homosynaptic depression	Restored^*∗*^	NA	Restored^*∗*^	NA	Restored^*∗*^	NA
Presynaptic inhibition of Ia afferents	No change	Not tested	Restored^*∗*^	↓ stance phase^*∗*^ ↑ swing phase^*∗*^	Restored^*∗*^	↓ late stance^*∗*^ ↑ swing-to-stance transition ^*∗*^
Reciprocal Ia inhibition	Restored^*∗*^	Not tested	Restored^*∗*^	↓ stance phase^*∗*^ ↑ swing phase^*∗*^	Restored^*∗*^	↓ stance phase^*∗*^ ↓ swing phase^*∗*^
Nonreciprocal Ib inhibition	Restored^*∗*^	Not tested	No change	No change in stance^*∗*^ ↓ early swing^*∗*^ ↑ late swing^*∗*^	Restored^*∗*^	↓ stance phase^*∗*^
Long-latency flexor reflexes	↓ R-Leg^*∗*^ ↑ L-Leg^*∗*^	Improved^*∗*^	↓ R-Leg^*∗*^ ↑ L-Leg^*∗*^	Improved^*∗*^	↓ R-Leg^*∗*^ ↑ L-Leg^*∗*^	Improved^*∗*^
Short-latency flexor reflexes	Reappeared in R-Leg^*∗*^	Phase-dependent modulation emerged in R-Leg^*∗*^	Reappeared in R-Leg^*∗*^	Phase-dependent modulation emerged in R-Leg^*∗*^	Reappeared in both legs^*∗*^	Phase-dependent modulation emerged^*∗*^

**(b):** 

Neuronal activity	AIS A/B		AIS C		AIS D	
	Seated	Standing	Seated	Standing	Seated	Standing
Soleus motoneuron excitability (Hmax)	No change in R/L-Legs	No change in R/L-Legs	No change in R-Leg^*∗*^ ↓ L-Leg^*∗*^	↑ R/L-Legs^*∗*^	↓ R-Leg^*∗*^ No change in L-Leg^*∗*^	No change in R/L-Legs
H-threshold	No change in R/L-Legs	No change in R/L-Legs	No change in R/L-Legs	No change in R/L-Legs	↑ R-Leg^*∗*^ No change in L-Leg^*∗*^	↓ L-Leg^*∗*^ No change in R-Leg^*∗*^

^#^Neuronal activity changes after locomotor training in people with SCI are from authors' recent published work [21, 54, 72, 90, 141]. R: right; L: left; and NA: not applicable; symbols ↑/↓ indicate increased or decreased neuronal activity. *∗* refers to neuronal activity changes.

## Abbreviations

AIS: American Spinal Injury Association Impairment Scale
BWS: Body weight support
CNS: Central nervous system
CPGs: Central pattern generators
C-T interval: Conditioning-test interval
EMG: Electromyograms/electromyography
EPSPs: Excitatory postsynaptic potentials
Hmax: Maximal H-reflex
MG: Medialis gastrocnemius
MEP: Motor evoked potential
PAD: Primary afferent depolarization
SCI: Spinal cord injury
TA: Tibialis anterior
TMS: Transcranial magnetic stimulation.
